# Supplementary material for: PD-1 antibody-bound progenitor-exhausted CD8+ T cells in lymph nodes boost PD-1-blockade anti-tumor immunity in gastrointestinal cancer
Source: Nat Commun. 2026 Apr 8;17:4969. doi: 10.1038/s41467-026-70751-2 (PMC13237225; doi:10.1038/s41467-026-70751-2)
Supplement: Supplementary file 1 — Supplementary Information [file 41467_2026_70751_MOESM1_ESM.pdf]

Supplementary Information

- **Title:** PD-1 antibody–bound progenitor-exhausted $CD8^+$ T cells in lymph nodes boost PD-1–blockade anti-tumor immunity in gastrointestinal cancer

- **First Author:** Yohei Nose, Yoshiaki Yasumizu
- **Corresponding Author:** Takuro Saito

Supplementary Fig. 1

	Sequencing analysis	Flow cytometry analysis
With ICI treatment	<u>**Figure 1**</u><br>***scRNA-seq analysis***<br>• ICI-treated patients with EGJ cancer (Patient 1,2)<br><u>**Figure 5, 6**</u><br>***scRNA/TCR/CITE-seq analysis***<br>• ICI-treated patients with EGJ (Patient 1,2) and gastric cancer (Patient 3)	<u>**Figure 4**</u><br>***Flow cytometry analysis***<br>• ICI-treated patients with gastrointestinal cancers (14 patients)
Without ICI treatment	• ICI-naive patients with gastric cancer (Patient 4,5)<br><u>**Figure 2**</u><br>***NMF analysis, Survival analysis in tumor***<br>• ICI-naive patients with gastric cancer from TCGA database	<u>**Figure 3**</u><br>***Flow cytometry analysis, Survival analysis***<br>• ICI-naive patients with gastric cancer (55 patients)

Supplementary Fig. 1. An overview of the study summarizing the experimental methods and sample details for each figure.

The details of experimental methods and sample details used in each figure are described, and the connections between the figures are highlighted.

Supplementary Fig. 2

a

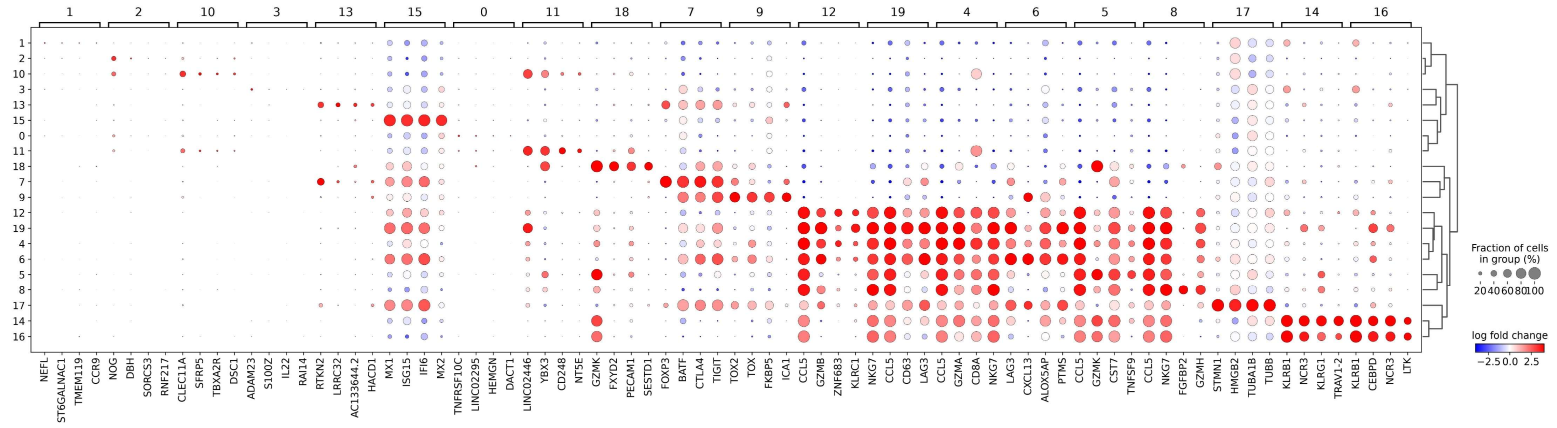

b

Cluster	Name	Representative genes	PBMC	LN	Tumor /Meta
**C00**	**CD4-naive**	**CD4, CCR7, TCF7, NR4A1**	**-**	**+++**	**-**
**C01**	**CD4-Tcm**	**CD4, CCR7, TCF7, CXCR5, GATA3**	**+++**	**+**	**+**
**C02**	**CD4-naive**	**CD4, CCR7, TCF7, NOG**	**+++**	**-**	**-**
**C03**	**CD4-Tcm**	**CD4, CCR7, TCF7, CXCR5, GATA3, NR4A1**	**-**	**+++**	**+**
**C04**	**CD8-Tex**	**CD8A, PDCD1, TOX, ENTPD1, ITGAE, TOX, CXCL13**	**-**	**+**	**+++**
**C05**	**CD8-Tpex**	**CD8A, TCF7, PDCD1, CXCL13, CXCR5, GZMK, NR4A1, TOX**	**-**	**+++**	**+**
**C06.0**	**CD8-Tex**	**CD8A, PDCD1, TOX, ENTPD1, ITGAE, TOX, CXCL13, ISG15**	**-**	**+**	**+++**
**C06.1**	**CD4-Tex**	**CD4, PDCD1, TOX, ENTPD1, TOX**	**-**	**+**	**+++**
**C07**	**CD4-TregEff**	**CD4, FOXP3, CTLA4, CCR8, BATF, PDCD1, ENTPD1**	**-**	**++**	**+++**
**C08**	**CD8-effector**	**CD8A, GZMK, CX3CR1, GNLY, FGFBP2**	**+++**	**-**	**+**
**C09**	**CD4-Tfh**	**CD4, TCF7, PDCD1, CXCL13, CXCR5, TOX, BCL6**	**-**	**+++**	**+**
**C10**	**CD8-naive**	**CD8A, CCR7, TCF7, LINC02446**	**+++**	**-**	**-**
**C11**	**CD8-naive**	**CD8A, CCR7, TCF7, LINC02446, NR4A1**	**-**	**+++**	**-**
**C12**	**CD8-Tpex**	**CD8A, TCF7, PDCD1, GZMK, NR4A1, TOX**	**-**	**+**	**+++**
**C13**	**CD4-Treg**	**CD4, FOXP3, CTLA4, CCR8, BATF**	**-**	**+++**	**+**
**C14**	**CD8-MAIT**	**CD8A, SLC4A10, GZMK, NR4A1**	**-**	**++**	**+++**
**C15**	**CD4-naive**	**CD4, CCR7, TCF7, MX1, MX2, ISG15, IFI6**	**-**	**+++**	**+**
**C16**	**CD8-MAIT**	**CD8A, GZMK, SLC4A10**	**+++**	**-**	**-**
**C17.0**	**CD8-Tex**	**CD8A, PDCD1, TOX, ENTPD1, ITGAE, TOX, CXCL13, ISG15, STMN1**	**-**	**+**	**+++**
**C17.1**	**CD8-Tex**	**CD8A, PDCD1, TOX, ENTPD1, ITGAE, TOX, CXCL13, ISG15, STMN1**	**-**	**+**	**+++**
**C17.2**	**CD4-Tex**	**CD4, PDCD1, TOX, ENTPD1, TOX, STMN1**	**+**	**++**	**+++**
**C18**	**doublet**	**-**	**+**	**+++**	**-**
**C19**	**CD8-Tex**	**CD8A, PDCD1, TOX, ENTPD1, ITGAE, TOX, CXCL13, LINC02446**	**-**	**-**	**+++**

Supplementary Fig. 2. Signature gene expression of each cluster in single-cell RNA-seq from two surgically resected esophagogastric junction cancers after ICI treatment.

a Dot plot depicting signature genes' mean expression levels and percentage of cells expressing them across clusters. **b** Overview of T cell cluster characteristics. The tissue distribution was categorized as -/+/++/+++.

Supplementary Fig. 3

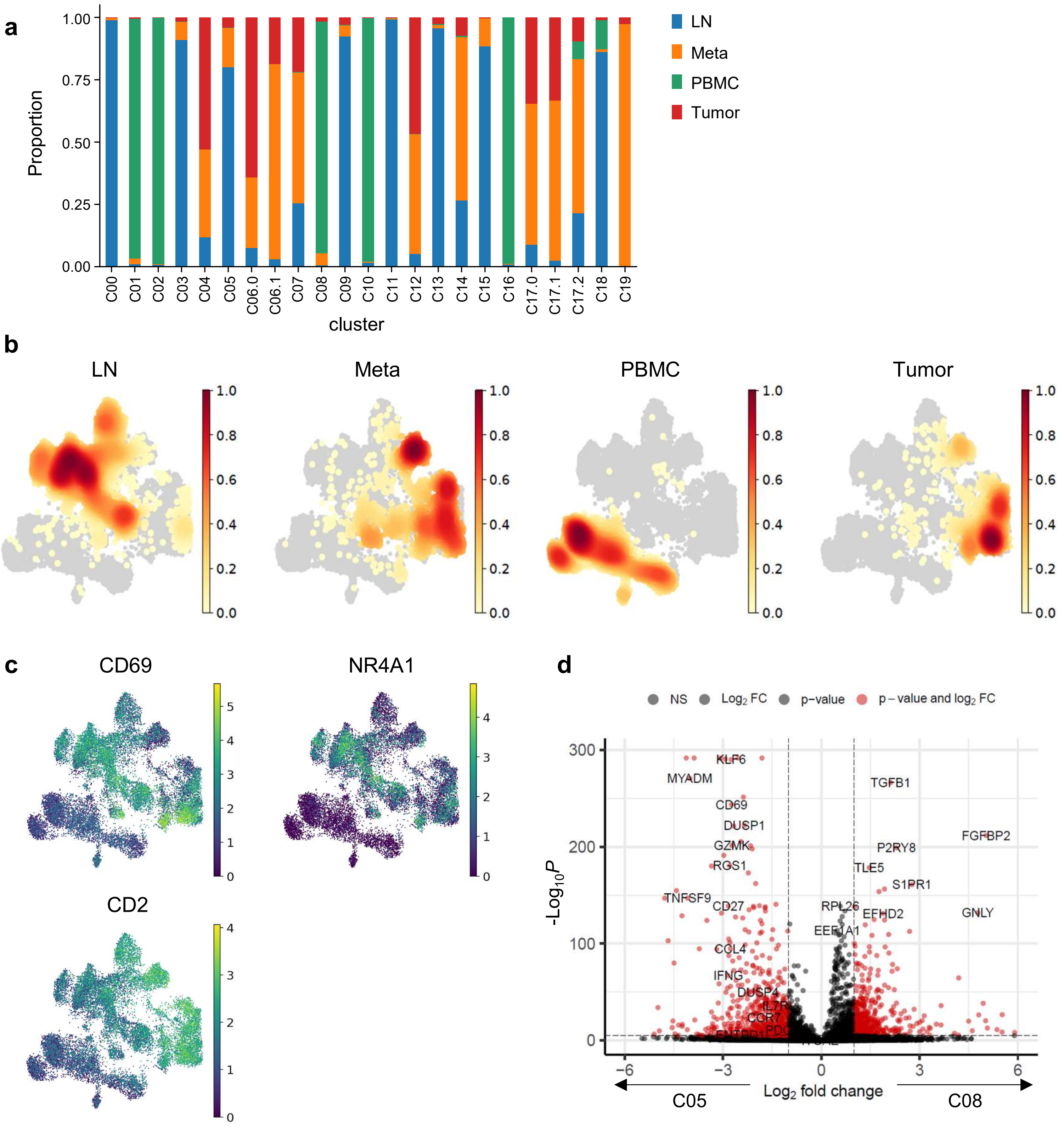

Supplementary Fig. 3. Tissue distribution of each cluster and differences in expressed genes between lymph nodes-enriched C05 and blood-enriched C08.

a The tissue distribution for each cluster in single-cell RNA-seq from two surgically resected esophagogastric junction cancers after ICI treatment. Liver metastases were defined as "Meta", and metastatic and metastasis-free LN as "LN". **b** Density plot showing T cell distribution from each tissue. **c** UMAP plot showing *CD69*, *NR4A1*, and *CD2* genes. All of these genes showed low expression levels in PBMCs, indicating differential expression compared to non-PBMC tissues. **d** Volcano plot showing the differentially expressed genes between the C05 (LNs) and C08 (PBMC) clusters.

Supplementary Fig. 4

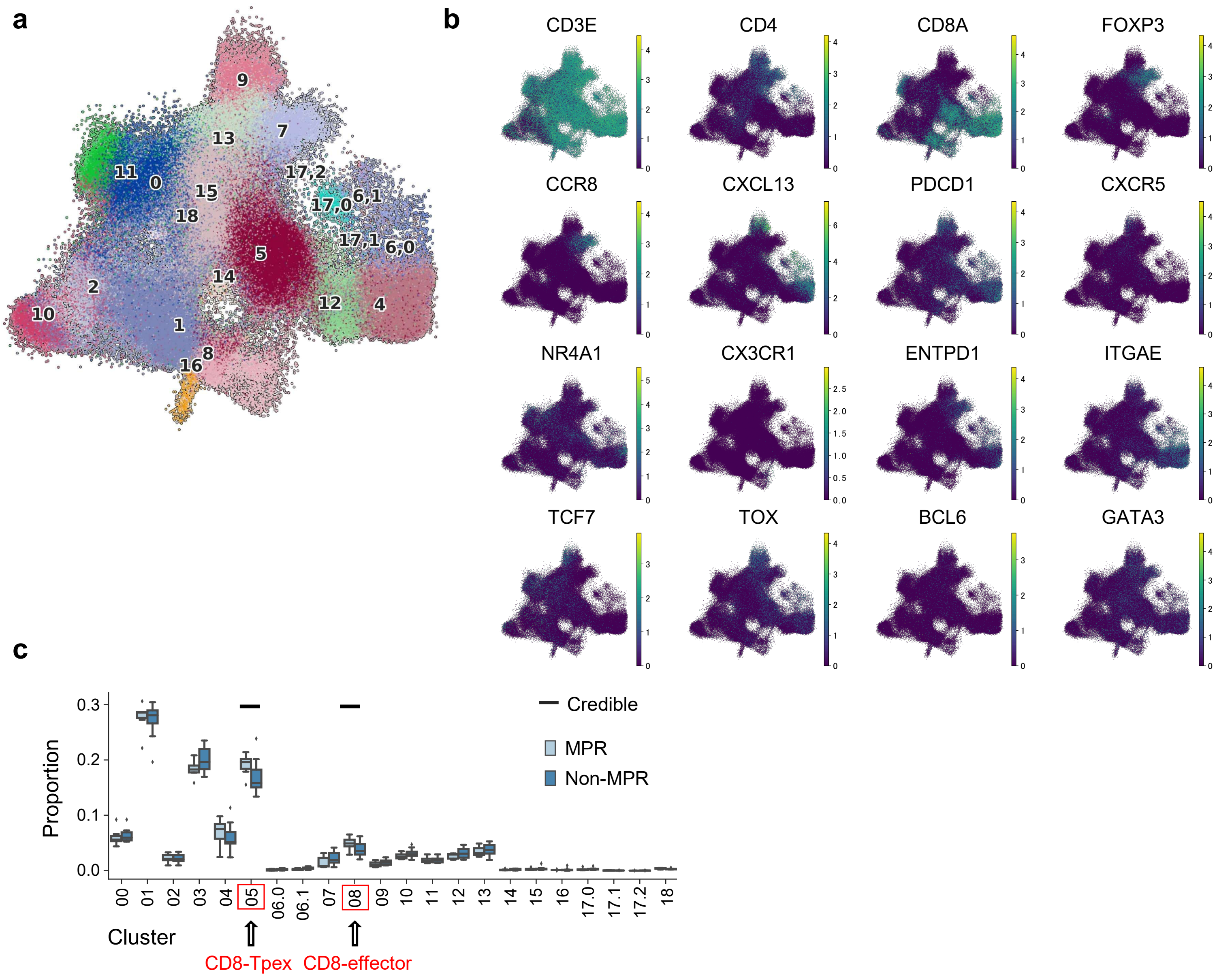

Supplementary Fig. 4. Single-cell RNA-seq profiling of T cells in lung cancers with neoadjuvant anti–PD-1 treatment.

a Uniform manifold approximation and projection (UMAP) projection of a previously reported single-cell RNA-seq dataset (GSE173351) of T cells of the tumor, adjacent normal lung, lymph nodes, and brain metastasis in lung cancers treated with neoadjuvant anti–PD-1. **b** UMAP projection of each marker gene. **c** Comparison of the proportion of each cluster between patients with the major pathologic response (MPR) and the non-major pathologic response (non-MPR). The data from a previous study (GSE173351) were used for the analysis. The single-cell RNA-seq data were projected on our reference using Symphony. A cell population that is considered significantly (FDR < 0.05) different in the single-cell compositional data analysis (scCODA) framework is described as credible.

Supplementary Fig. 5

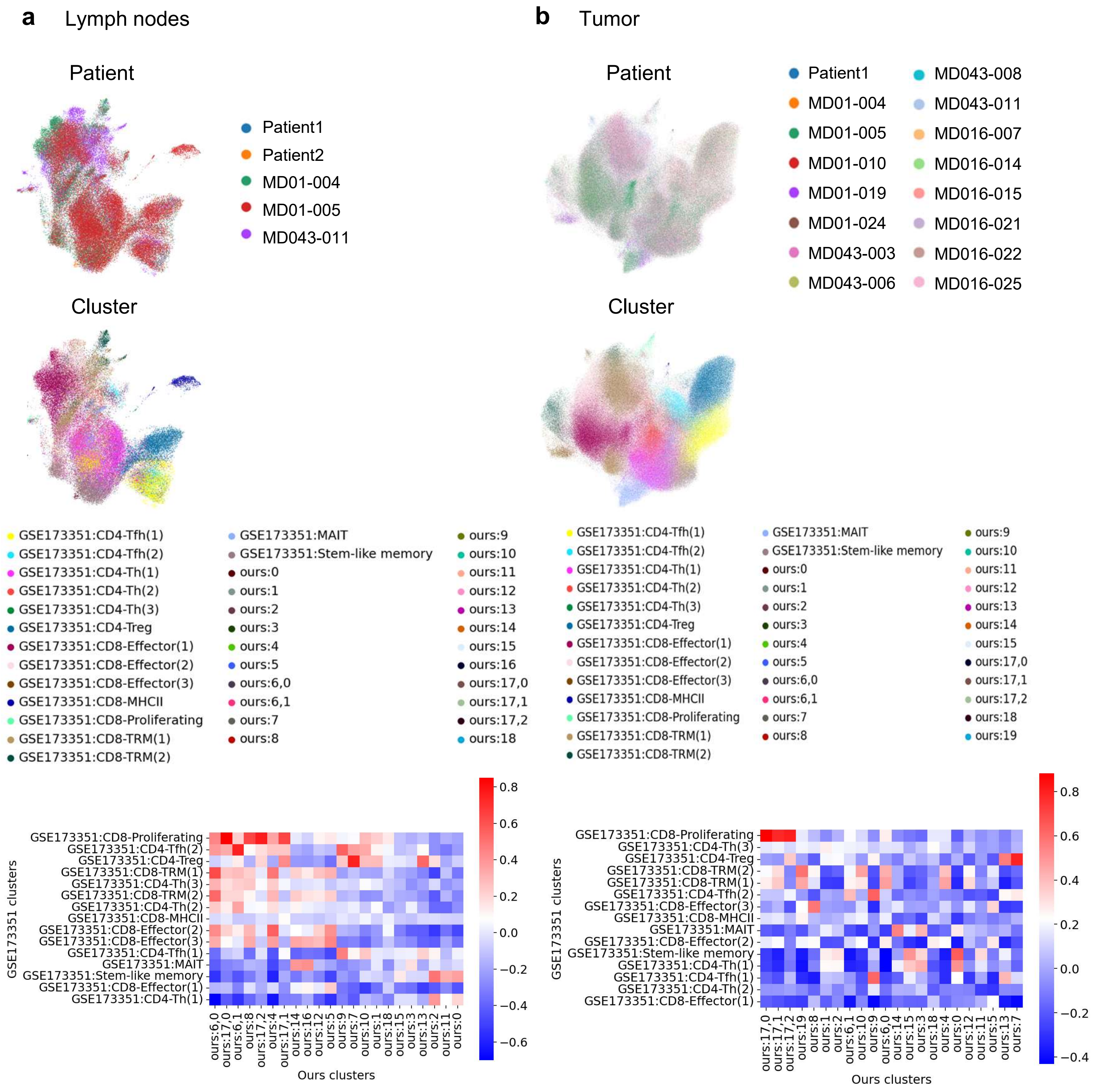

Supplementary Fig. 5. Comparison of our single-cell RNA-seq data and GSE173351 data in lymph nodes and tumor.

Uniform manifold approximation and projection (UMAP) plot of T cells in single-cell RNA-seq, colored by patients and clusters, and a heatmap comparing clusters per tissue in lymph nodes (a) and tumor (b).

Supplementary Fig. 6

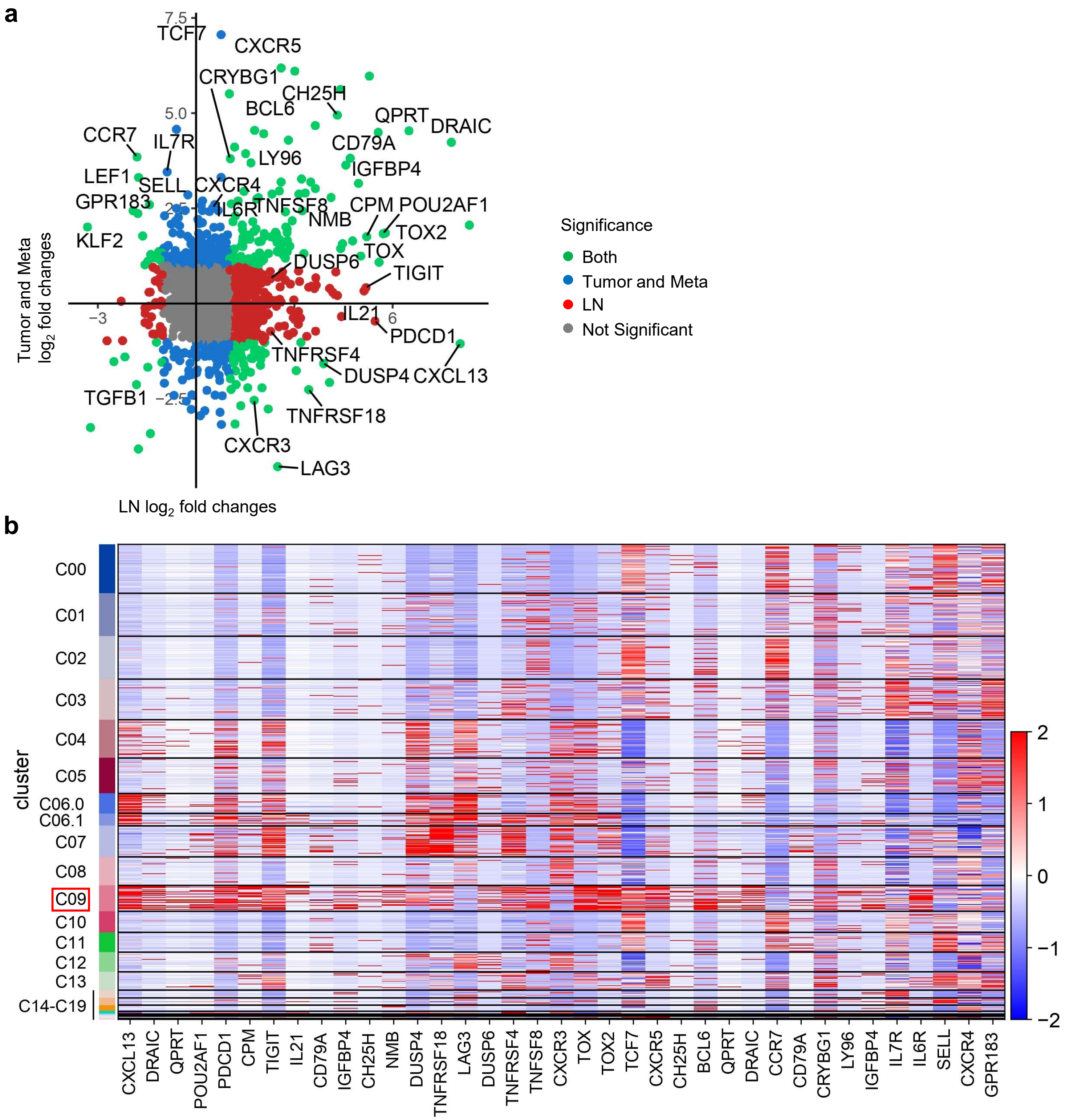

Supplementary Fig. 6. Transcriptional signature of CD4-Tfh in lymph nodes.

a Scatter plot of genes expressed differentially between CD4-Tfh (C09) cells in LNs and CD4-Tex (C06.1, C17.2) cells in tumor and liver metastases (represented by blue dots), and between CD4-Tfh (C09) cells in LNs and CD4-naive (C00) and CD4-Tcm (C03) cells in LNs (represented by red dots). Differentially expressed genes in both comparisons are shown in green dots. Genes with normalized expression > 0.3 in C09 are shown. P-values < 0.05 were considered significant. Two-sided tests were used for differential expression analysis. **b** Heatmap of differentially expressed genes from the same analysis as (**a**). The CD4-Tfh cluster (C09) is highlighted in a red box.

Supplementary Fig. 7

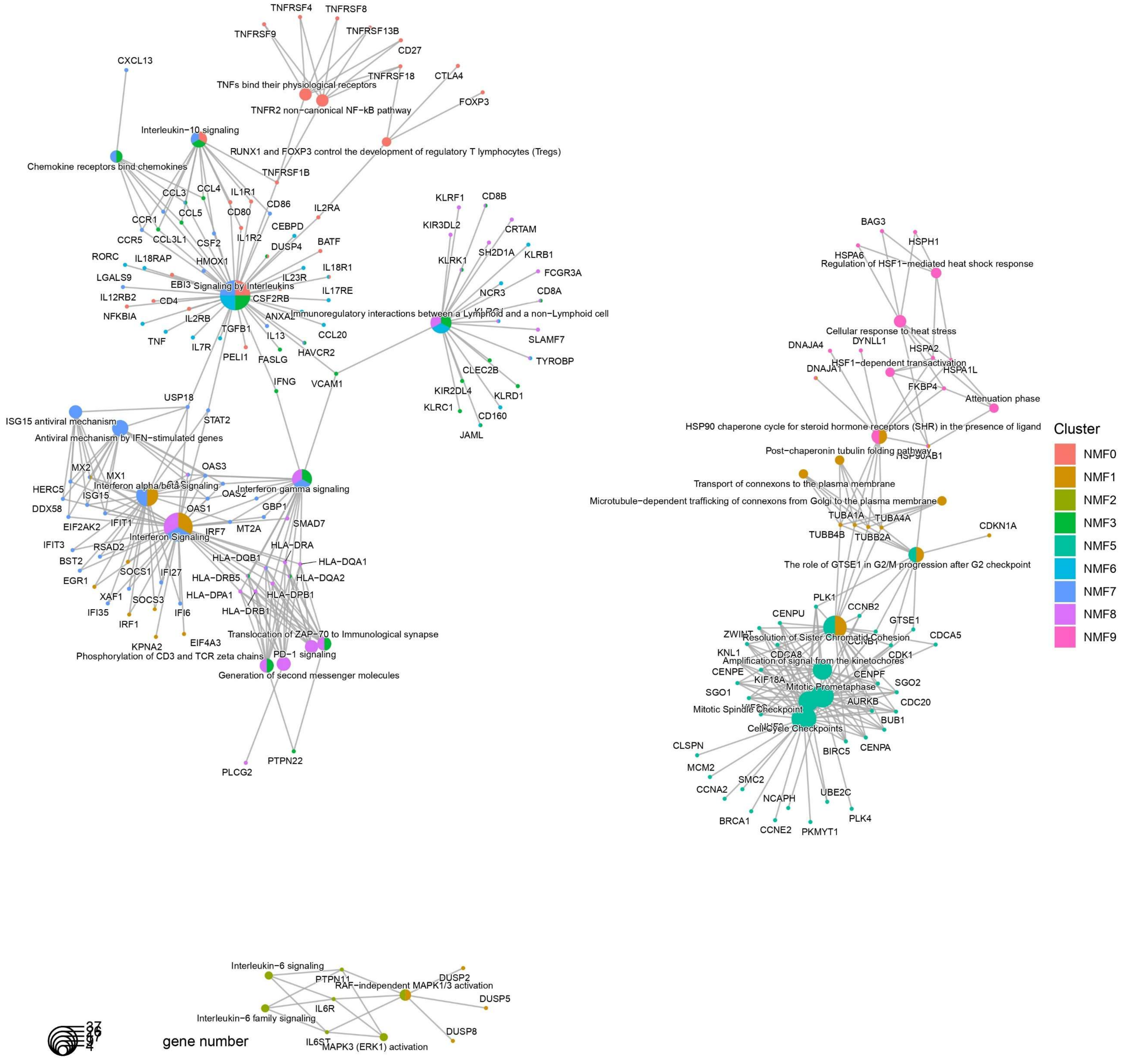

Supplementary Fig. 7. REACTOME analysis by NMF in the TCGA gastric cancer dataset.

Enriched REACTOME pathways and associated genes in each NMF. The node size represents the number of genes included in each pathway, and the color represents the adjusted P-value of the enrichment.

Supplementary Fig. 8

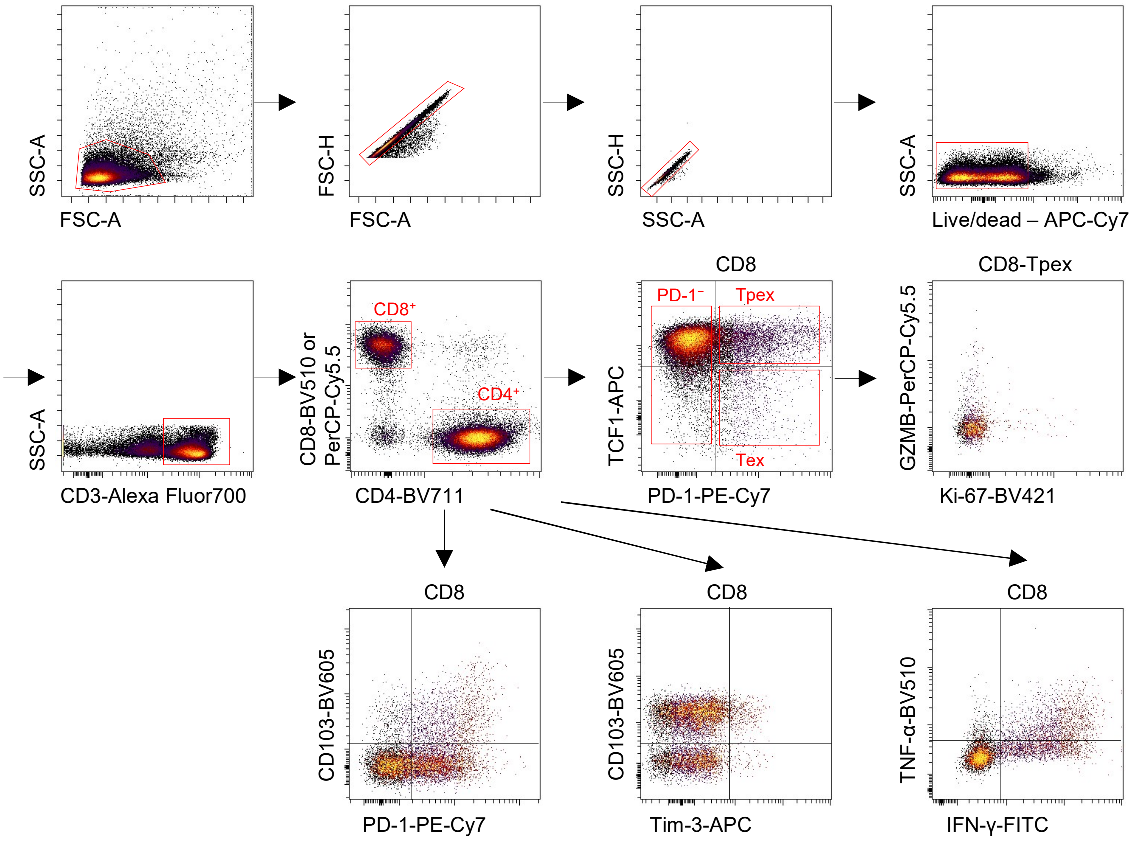

Supplementary Fig. 8. The gating strategy of flow cytometry.

According to the above, $CD3^+$ T cells were divided into $CD8^+$ and $CD4^+$ T cells, and their surface molecular expression was analyzed (Fig. 3b, g). Intracellular cytokine staining assay (IFN-γ, TNF-α) was performed (Supplementary Fig. 9). Furthermore, PD-1^+ T cells are subdivided into three subpopulations based on the presence or absence of TCF1 expression, as described in the previous reports: Tpex ($TCF1^+$PD-1^+ T cells), Tex ($TCF1^-$PD-1^+ T cells), and PD-1^- T cells (Fig. 3d, h). The expression of Ki-67 and GZMB in each of these subpopulations was then examined (Fig. 3e, i, 4d-h).

Supplementary Fig. 9

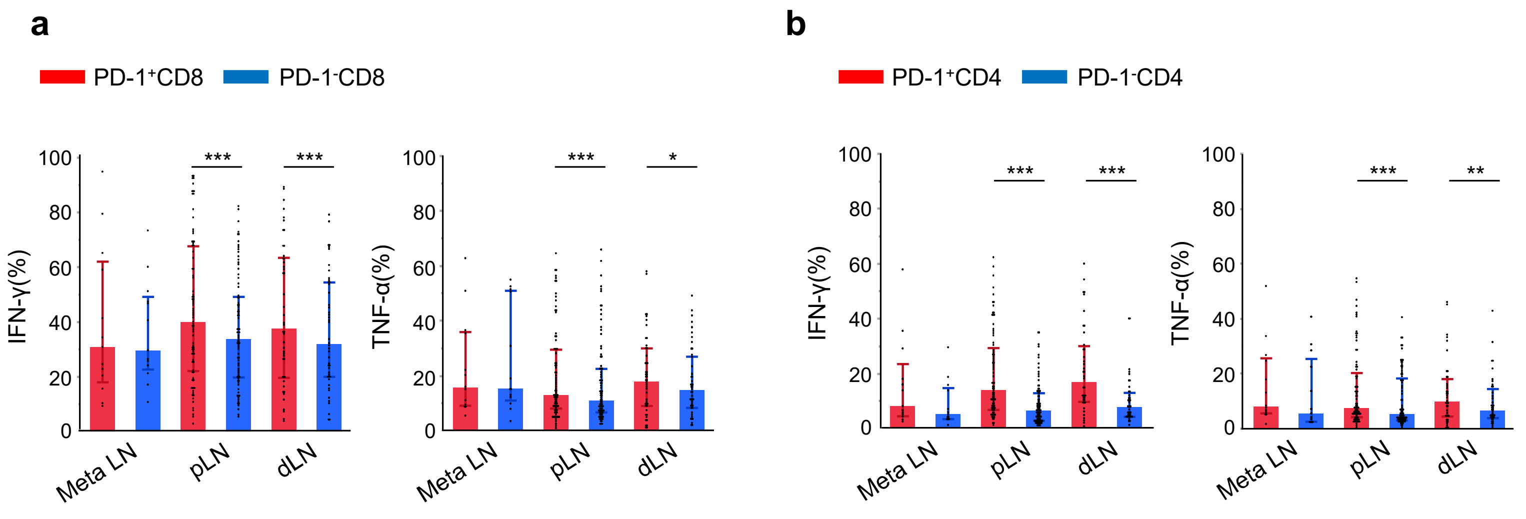

Supplementary Fig. 9. Cytokine production by PD-1⁺ and PD-1⁻ T cells in each tissue of ICI-naive gastric cancer patients.

The frequency of IFN-γ⁺ (left) and TNF-α⁺ (right) among PD-1⁺ or PD-1⁻ CD8⁺ T cells **(a)** and CD4⁺ T cells **(b)** by flow cytometry analysis according to tissue types (CD8: Meta LN: n=13, pLN: n=83, dLN: n=46/ CD4: Meta LN: n=13, pLN: n=81, dLN: n=40). Error bars indicate the median ± interquartile range. The significance of differences in the frequency of IFN-γ⁺ and TNF-α⁺ cells between PD-1⁺ and PD-1⁻ T cells per tissue was calculated using the nonparametric Wilcoxon matched-pairs signed-rank test (*$P < 0.05$, **$P < 0.01$, ***$P < 0.001$). Meta LN: metastatic lymph node, pLN: proximal lymph node, dLN: distal lymph node.

Supplementary Fig. 10

a Tumor

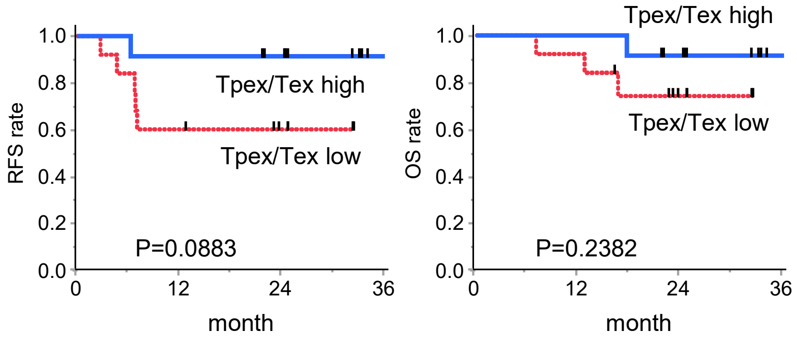

b Metastatic lymph nodes

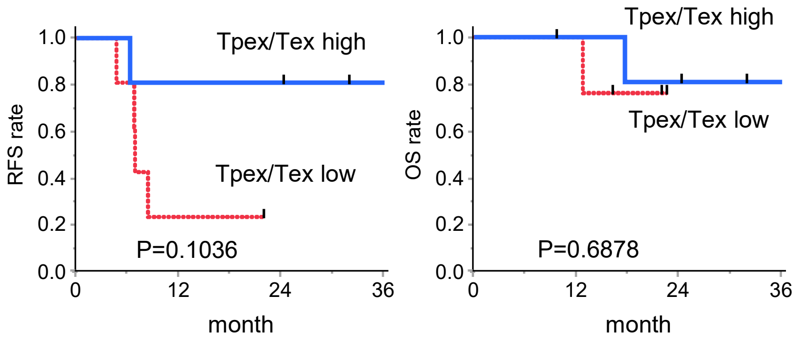

Supplementary Fig. 10. Kaplan-Meier curves for ICI-naive gastric cancer patients with high and low CD8-Tpex/Tex ratio in tumor and metastatic lymph nodes.

a Kaplan–Meier curves for recurrence-free survival rate (RFS) and overall survival rate (OS) were compared between high and low groups of the Tpex/Tex ratio in tumor (high: n=11, low: n=12). The Tpex/Tex ratio was calculated as the ratio of TCF1^{+}PD-1^{+} to TCF1^{-}PD-1^{+} among CD8^{+} T cells and divided into two groups based on the median value. **b** Kaplan–Meier curves for RFS and OS were compared between high and low groups of the Tpex/Tex ratio in metastatic lymph nodes (high: n=5, low: n=5). **a, b**, Differences were assessed using the log-rank test.

Supplementary Fig. 11

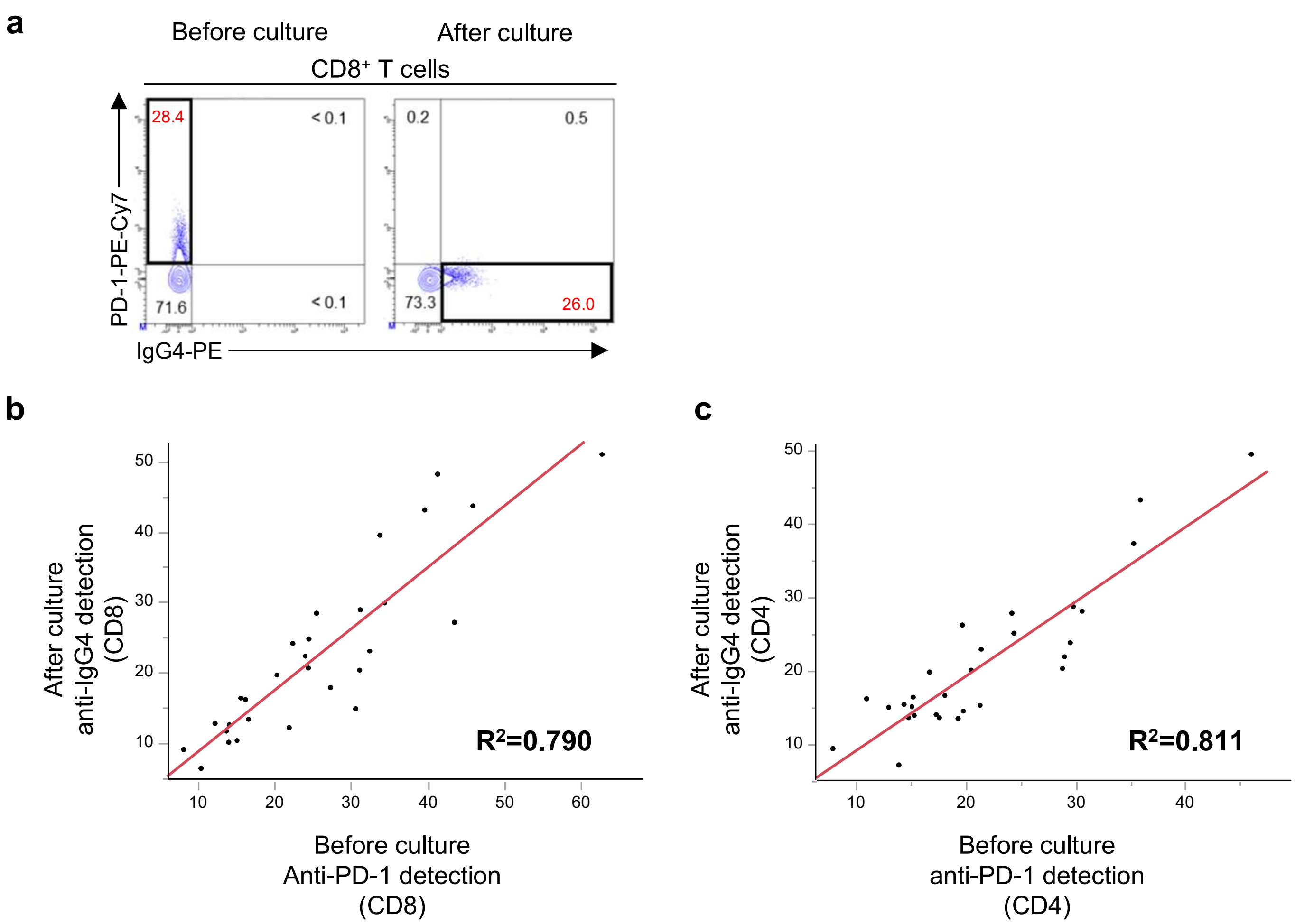

Supplementary Fig. 11. The accuracy of anti-IgG4 antibodies staining method in flow cytometry.

a Representative staining of $CD8^+$ T cells with anti-PD-1 and anti-IgG4 antibodies before and after in vitro culture with anti-PD-1 therapeutic antibody. The $PD\text{-}1^+IgG4^-CD8^+$ T-cell fraction before *in vitro* culture and the $PD\text{-}1^-IgG4^+$ $CD8^+$ T-cell fraction after culture are indicated by black squares. **b, c** The proportion of anti–IgG4 antibody in $CD8^+$ T cells **(b)** and $CD4^+$ T cells **(c)** after in vitro culture with anti–PD-1 therapeutic antibodies was compared to that of anti-PD-1 antibody in T cells before culture, using PBMC samples from ICI-naive gastric cancer patients (n=29). The coefficient of determination (R^2) is calculated with the Analysis of Variance in Regression Analysis (ANOVA).

Supplementary Fig. 12

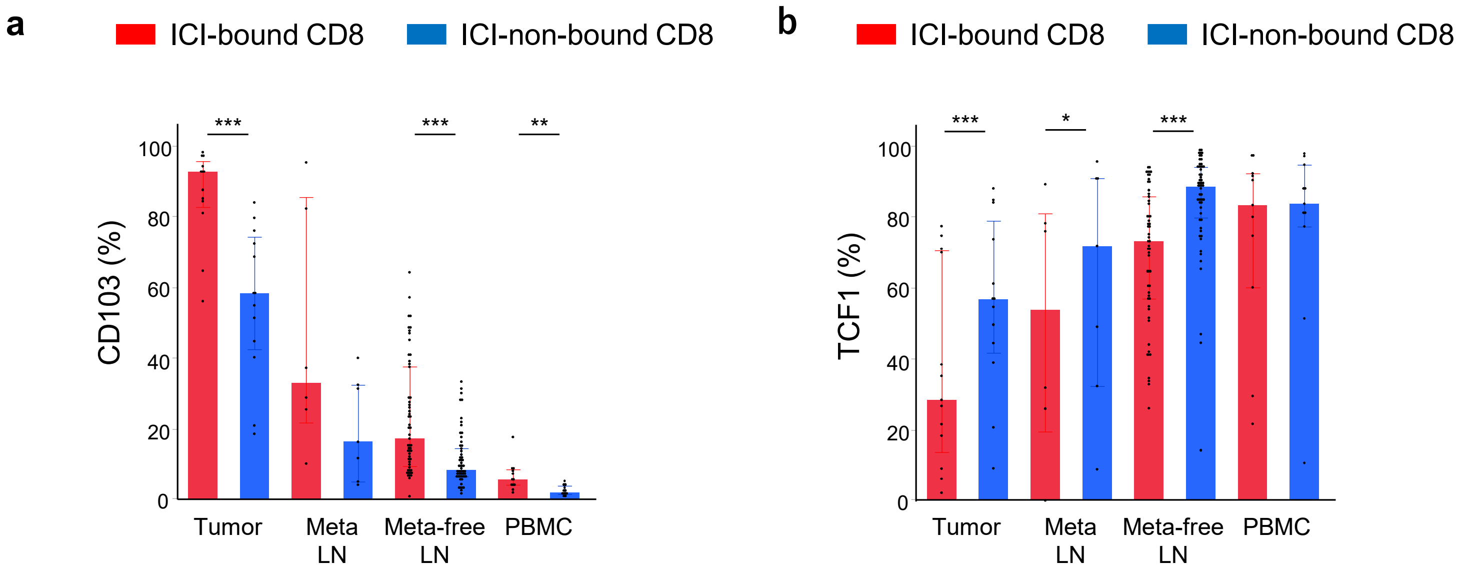

Supplementary Fig. 12. Comparison of marker expression between ICI-bound and ICI-non-bound CD8⁺ T cells in each tissue of ICI-treated patients by flow cytometry.

The frequency of CD103 **(a)** and TCF1 **(b)** expression among ICI-bound and ICI-non-bound $CD8^+$ T cells was shown in each tissue. Error bars indicate the median ± interquartile range. The significance of the differences between ICI-bound $CD8^+$ T cells and ICI-non-bound $CD8^+$ T cells in the same tissue was calculated using the nonparametric Wilcoxon matched-pairs signed-rank test (*P < 0.05, **P < 0.01, ***P < 0.001). Tumor: n=13, meta-LN (metastatic lymph node): n=6, meta-free LN (metastasis-free lymph node): n=55, PBMC: n=12.

Supplementary Fig. 13

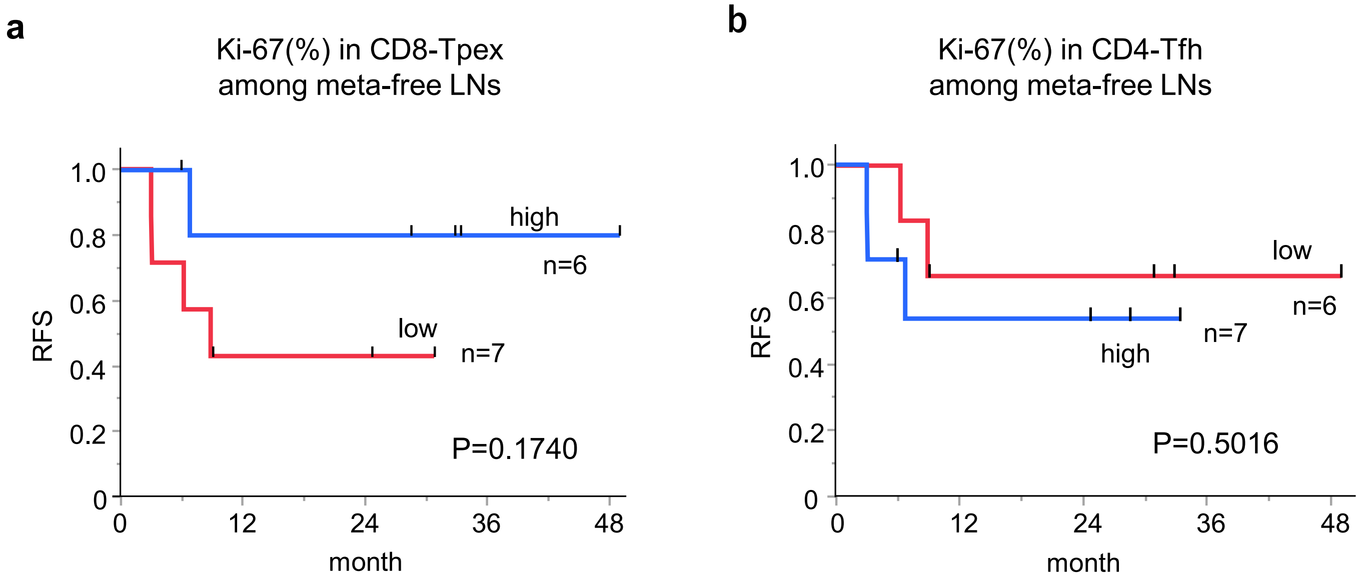

Supplementary Fig. 13. The association between Ki-67 expression levels in CD8-Tpex or CD4-Tfh cells within metastasis-free lymph nodes of ICI-treated patients and clinical outcomes.

Kaplan–Meier curves for recurrence-free survival rate (RFS) were compared between high and low groups of the Ki-67 percentage in CD8-Tpex **(a)** or CD4-Tfh **(b)** among metastasis-free lymph nodes (n=13). The two groups were divided based on the median value. P-values were calculated by using the log-rank test.

Supplementary Fig. 14

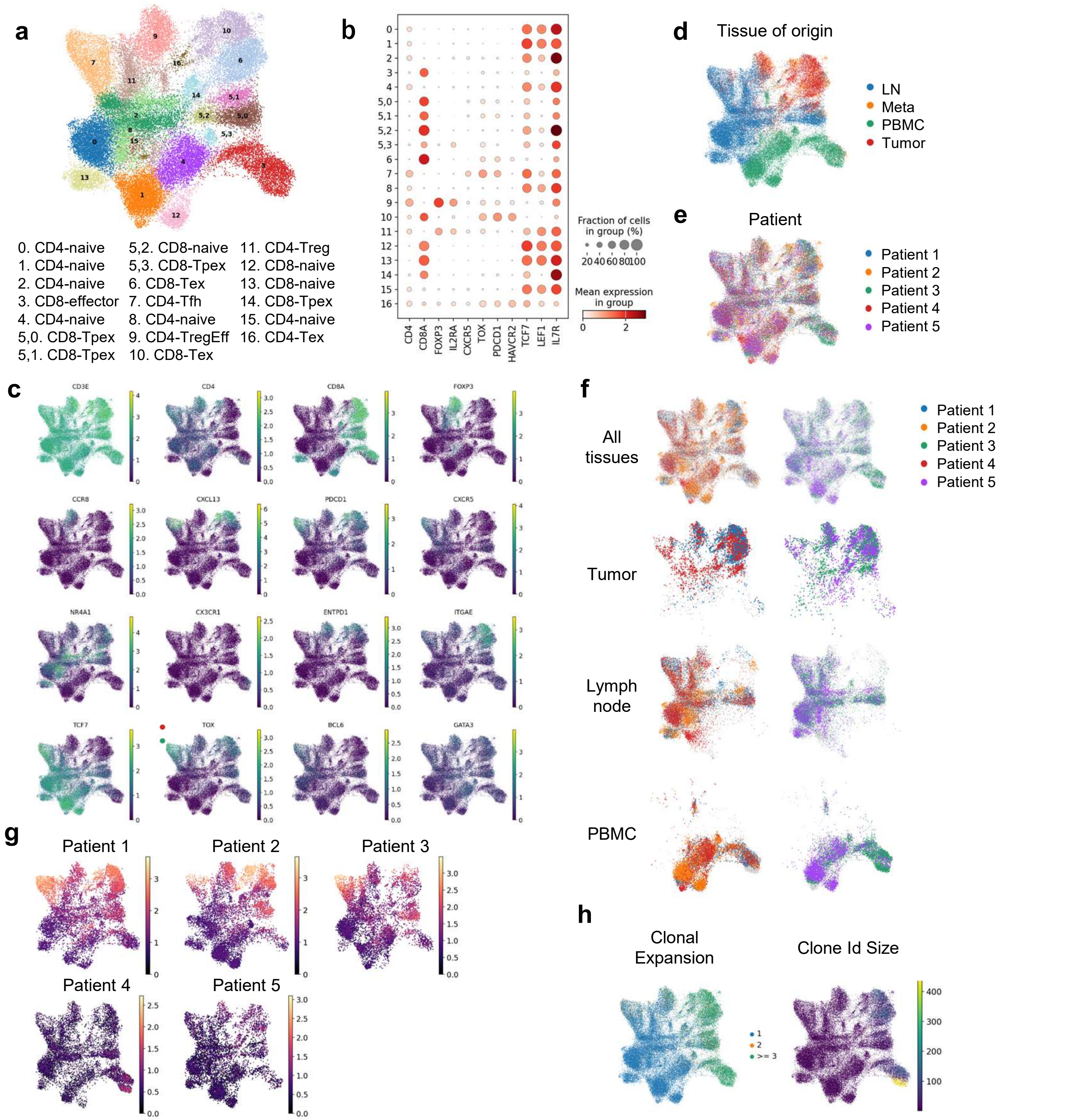

Supplementary Fig. 14. Single-cell RNA/TCR/CITE-seq in all 5 patients.

Uniform manifold approximation and projection (UMAP) plot of T cells in single-cell RNA-seq from all 5 patients, colored by Leiden cluster (**a**), marker genes (**c**), tissue of origin (**d**), and patient (**e**). **b** Dot plot showing the canonical gene expression per cluster. **f** UMAPs of individual tissues, shown separately for ICI-treated (left: patients 1, 2, 3) and ICI-naive (right: patients 4, 5) patients. The results for the three ICI-treated cases are shown on the left, and those for the two ICI-naive cases are shown on the right, separated by tissue type. **g** UMAP plot of bound IgG4 in single-cell RNA-seq from ICI-treated (patient 1,2,3) and ICI-naive patients (patient 4,5), respectively. **h** UMAP plot showing clonotype sizes in low-size categories (left) and actual numbers (right). The color indicates the number of cells that share the same T-cell receptor (TCR) sequences. In the left plot, clone sizes are specifically labeled to represent categories with clone sizes 1, 2, and greater than 3.

Supplementary Fig. 15

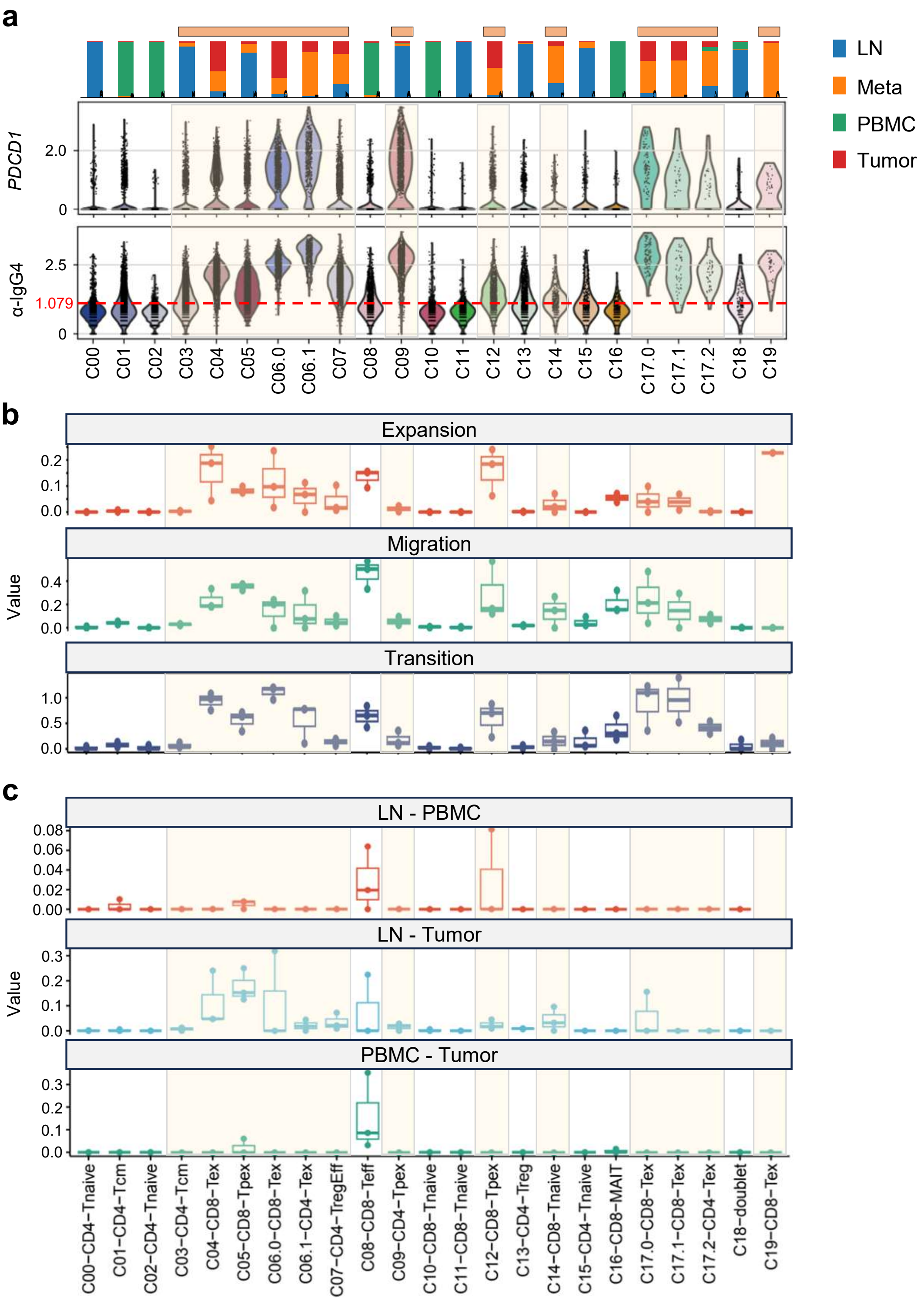

Supplementary Fig. 15. Single-cell RNA/TCR/CITE-seq analysis identified clonal expansion of ICI-bound CD8-Tpex after ICI treatment.

a Violin plots of the expression of *PDCD1* mRNA (top) and IgG4 (bottom) in each cluster. The tissue distribution for each cluster was shown at the top of the heatmap. The cutoff (1.079) is indicated by the red line. **b** Potentials of clonal expansion (top), tissue migration (middle), and developmental transition (bottom) of T cells in each cluster qualified by overall STARTRAC-expansion, migration, and transition indices. **c** Comparison of migration potentials of T cells in each cluster by pairwise STARTRAC-migration indices. **a-c** Clusters with high IgG4 binding are highlighted in orange boxes. **b, c** The box plots show the median (center line), the interquartile range (25th–75th percentiles; box), and the minimum to maximum values (whiskers).

Supplementary Fig. 16

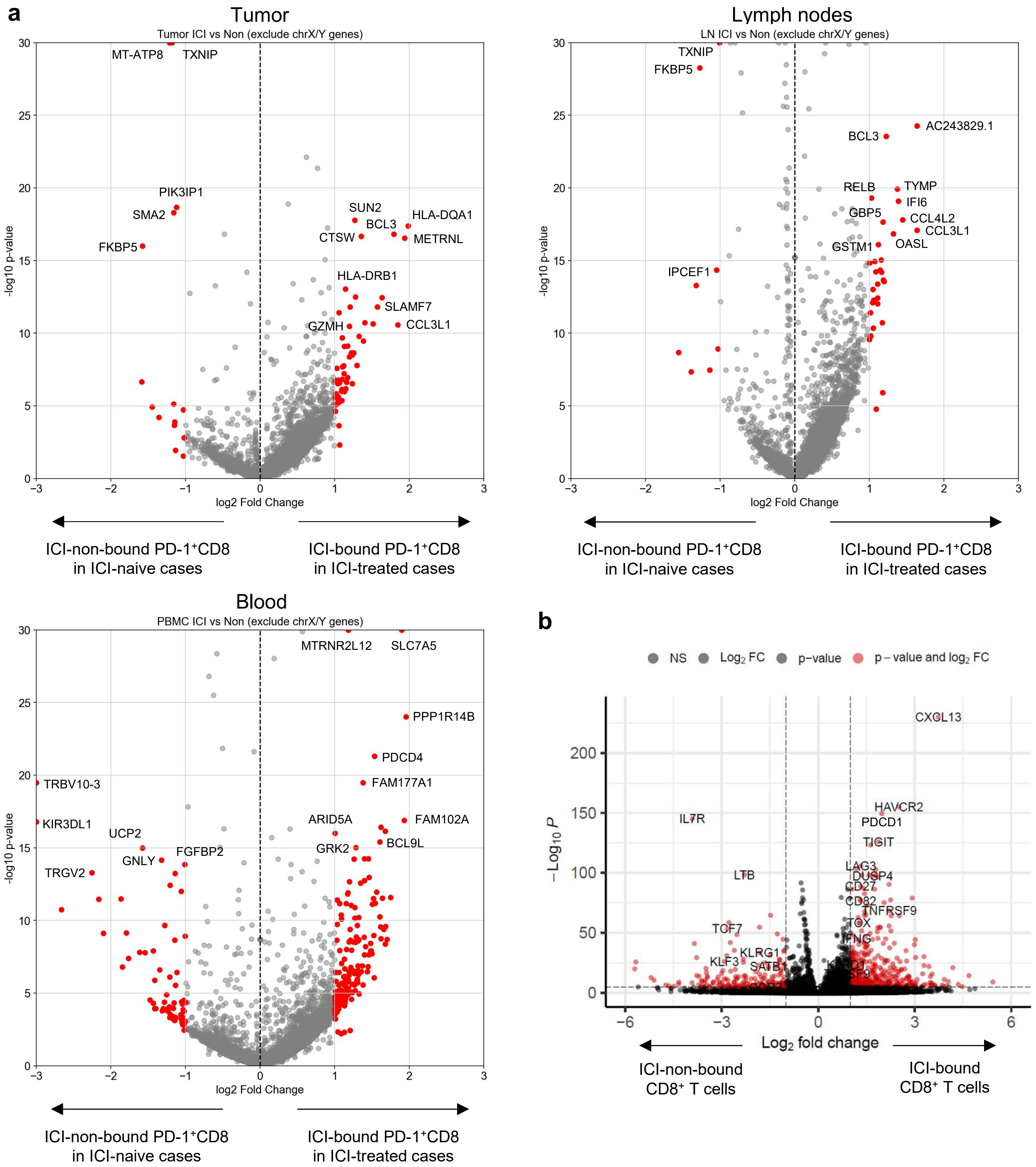

Supplementary Fig. 16. The characteristics of ICI-bound PD-1$^+$CD8$^+$ T cells in ICI-treated cases.

a Volcano plot showing the differences in gene expression between ICI-bound PD-1$^+$CD8$^+$ T cells in ICI-treated cases (patient 1, 2, 3) and ICI-non-bound PD-1$^+$CD8$^+$ T cells in ICI-naive patients (patient 4, 5) per tissue. Genes related to sex differences were excluded. **b** Volcano plot showing the differences of gene expression between ICI-bound and ICI-non-bound CD8$^+$ T cells in tumors from the three ICI-treated patients (patient 1, 2, 3).

Supplementary Fig. 17

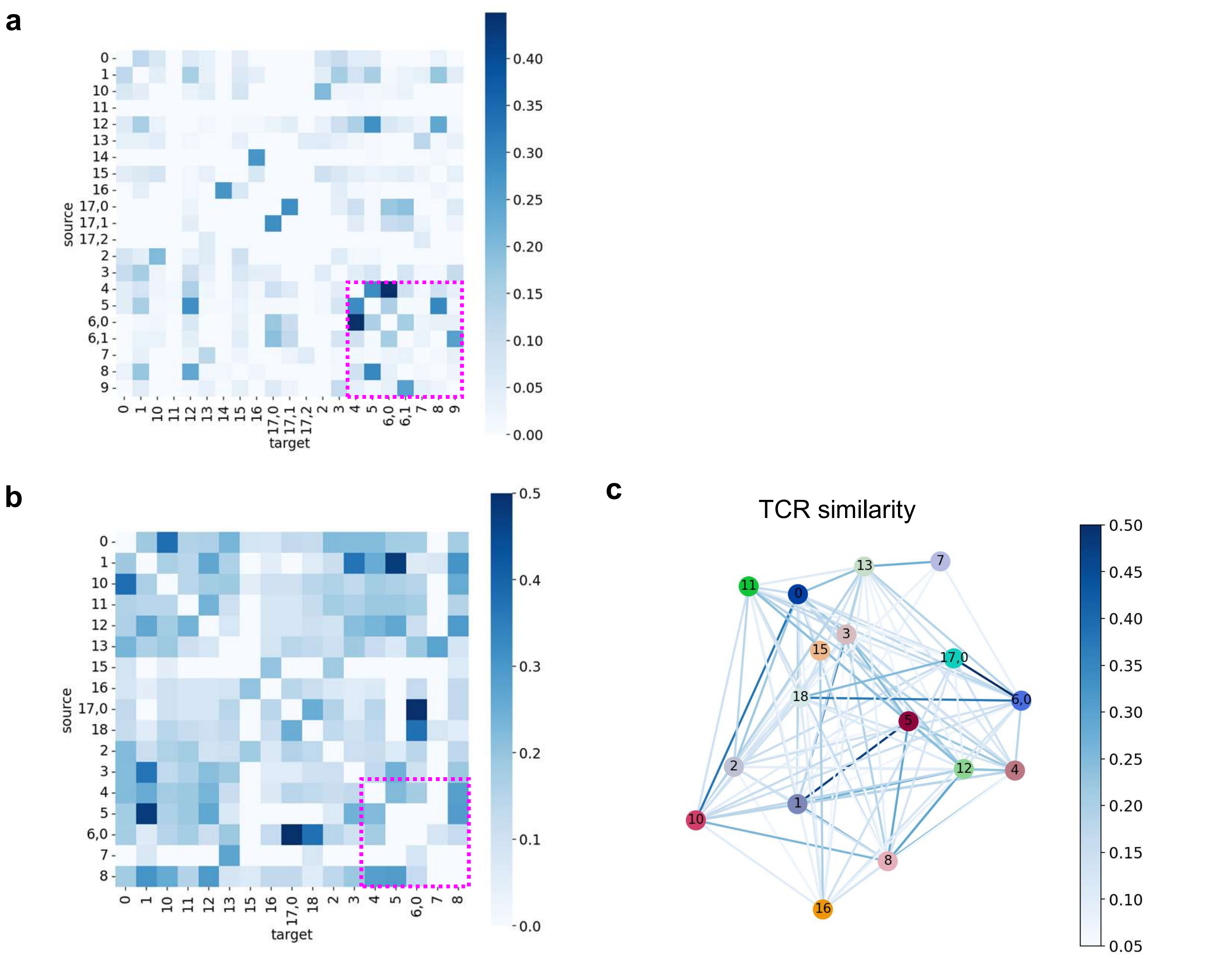

Supplementary Fig. 17. TCR similarity among clusters in ICI-treated and ICI-naive cases.

a Heatmap illustrating the TCR similarity among clusters in ICI-treated cases. **b** Heatmap illustrating the TCR similarity among clusters in ICI-naive cases. **c** TCR similarity network for each cluster in ICI-naive cases. TCR similarities were calculated for clones observed in Tumor, Meta, or LN. Jaccard Index is used for the similarity metric (see method). Edges with TCR similarity > 0.005 are shown. **b, c,** Clusters not detected in the ICI-naive cases were excluded from the analysis.

Supplementary Fig. 18

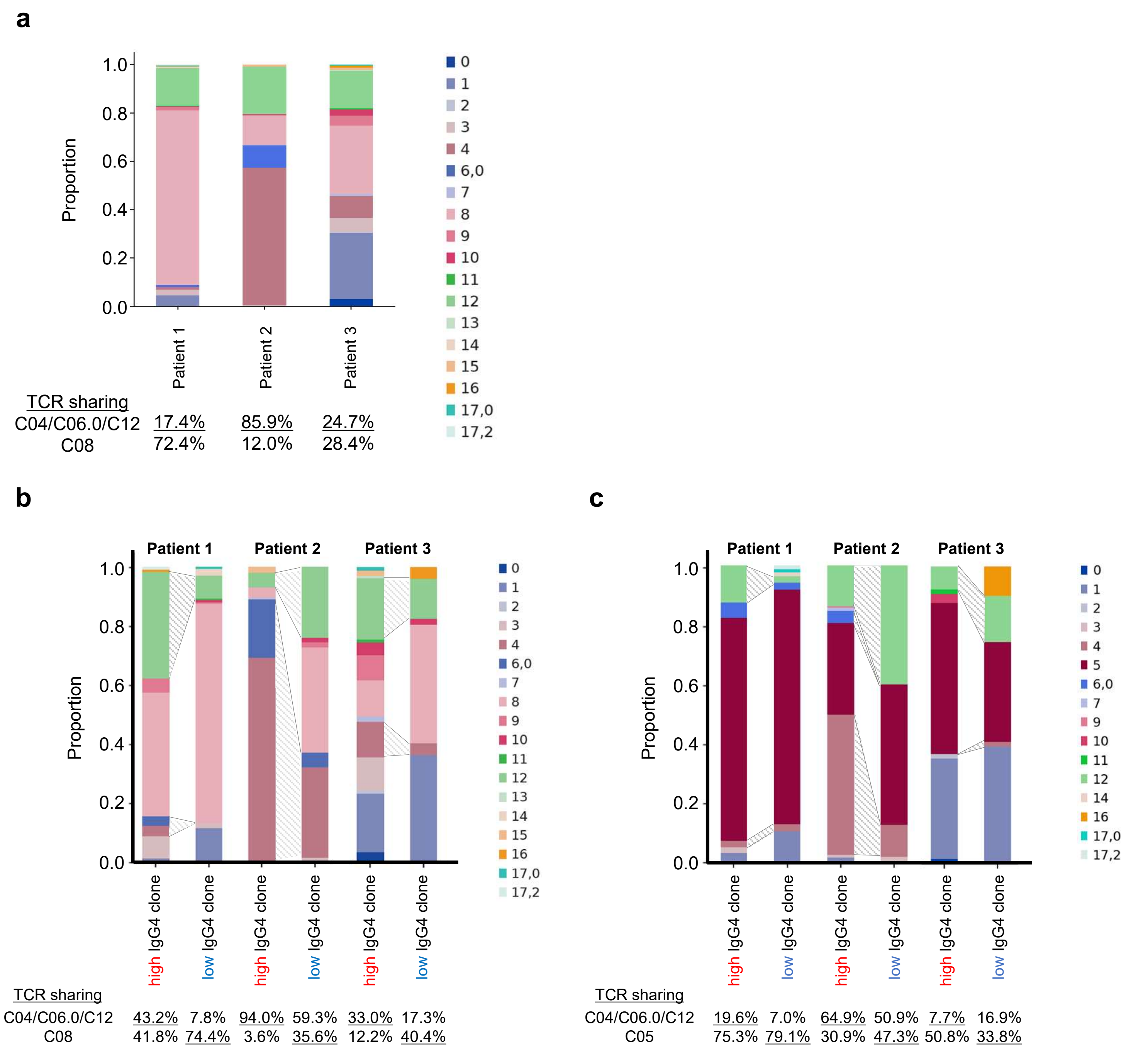

Supplementary Fig. 18. TCR sharing of high- and low-IgG4 clones in C05 and C08 with other clusters.

a Bar graph showing the percentage of TCR sharing between C05 and the other clusters in three ICI-treated patients (Patient 1, 2, 3). Clusters not shown indicate zero cells with TCR sharing. The percentage of TCR sharing is shown between cells in the C05 cluster and those in the C04, C06.0, and C12 clusters or the C08 cluster. **b** Bar graph showing the percentage of TCR sharing between the high- and low-IgG4 binding cells within the C05 cluster and those in the other clusters per patient. The percentage of TCR sharing is shown between high- or low-IgG4 cells in the C05 cluster and those in the C04, C06.0, and C12 clusters or the C08 cluster. **c** Bar graph showing the percentage of TCR sharing between the high and low-IgG4 binding cells within the C08 cluster and those in the other clusters per patient. The percentage of TCR sharing is shown between high- or low-IgG4 cells in the C08 cluster and those in the C04, C06.0, and C12 clusters or the C05 cluster. **b, c**, the CD8-Tex (C04, C06.0) and CD8-Tpex (C12) clusters are highlighted with oblique patterns and connected across patients.

Supplementary Fig. 19

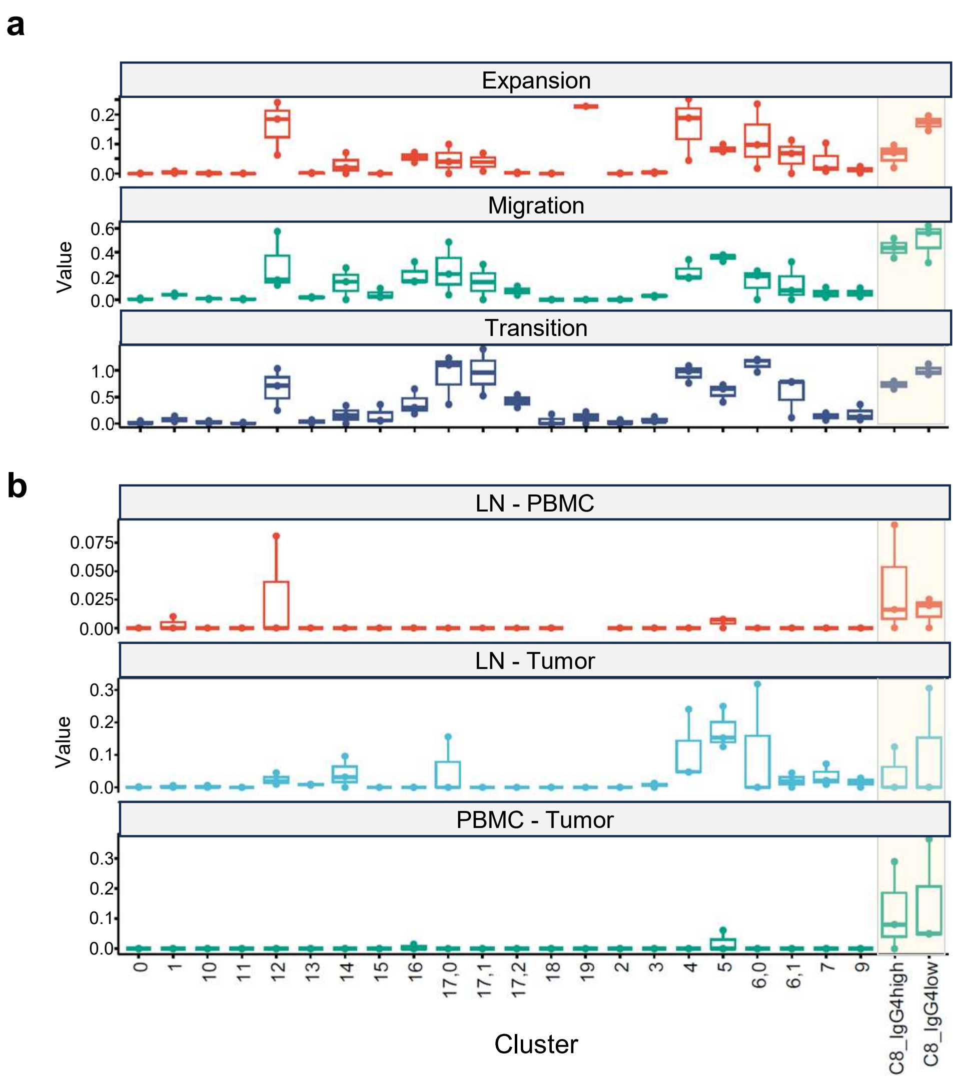

Supplementary Fig. 19. STARTRAC analysis of the high- and low-IgG4 cells within the C08 cluster in three ICI-treated patients.

a Potentials of clonal expansion (top), tissue migration (middle), and developmental transition (bottom) of T cells in each cluster qualified by overall STARTRAC-expansion, migration, and transition indices. **b** Comparison of migration potentials of T cells in each cluster by pairwise STARTRAC-migration indices. **a, b**, the high and low IgG4 binding groups in C08 are highlighted in orange boxes. The box plots show the median (center line), the interquartile range (25th–75th percentiles; box), and the minimum to maximum values (whiskers).

Supplementary Fig. 20

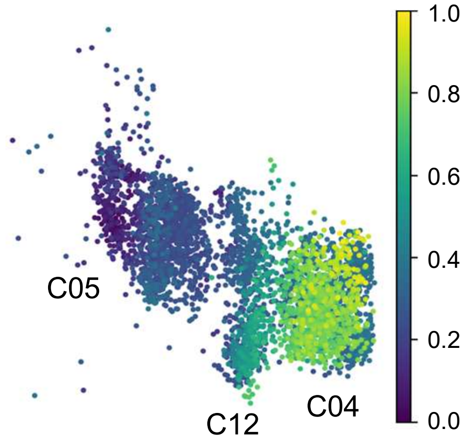

Supplementary Fig. 20. Pseudotime analysis of CD8⁺ T cells in lymph nodes and tumor.

Pseudotime analysis of T cells among the C04 (CD8-Tex), C05 (CD8-Tpex), and C12 (CD8-Tpex) clusters. Pseudotime was calculated using sc.tl.dpt.

Supplementary Fig. 21

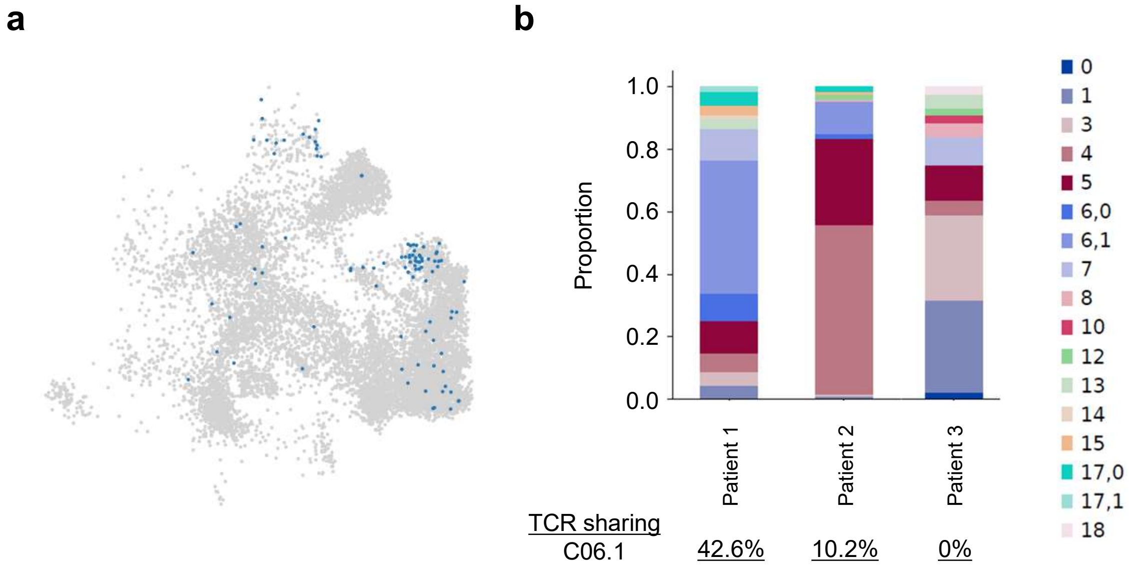

Supplementary Fig. 21. TCR sharing of clones in CD4-Tfh (C09) with other clusters.

a UMAP plot showing the clones in the tumor/meta compartments that are shared with CD4-Tfh in LNs (C09), which are indicated by blue dots. The dots represent only the tumor/meta compartments. **b** The bar graph showing the percentage of TCR sharing between C09 and the other clusters in three ICI-treated patients (Patient 1, 2, 3). Clusters not shown indicate zero cells with TCR sharing. The percentage of TCR sharing is shown between cells in the C09 cluster and those in the C06.1 cluster.

Supplementary Table 1. Clinicopathological characteristics of patients for sequencing analysis.

(a) Clinicopathological characteristics of two ICI-treated patients for initial single-cell RNA-seq analysis and single-cell RNA/TCR/CITE-seq analysis

Patient	Cancer type	Preoperative treatment	cTNM*	(y)pTNM*	Pathological response	Sample details
1	EGJ*	XELOX* + Nivolumab	cT3N1M1	ypT3N2M0	Grade 1a	tumor, meta LN*, meta-free LN*, PBMC*
2	EGJ*	FP* + Pembrolizumab	cT3N2M1	ypT2N0M0	Grade 1a	liver metastases, meta-free LN, PBMC*

(b) Clinicopathological characteristics of one additional ICI-treated and two ICI-naive patients for single-cell RNA/TCR/CITE-seq analysis

Patient	Cancer type	Preoperative treatment	cTNM*	(y)pTNM*	Pathological response	Sample details
3	Gastric	FOLFOX* + Nivolumab	cT4bN1M0	ypT1bN0M0	Grade 2	tumor, two meta-free LNs*, PBMC*
4	Gastric	None	cT3N2M0	pT4aN3M0	NA	tumor, meta LN*, meta-free LN*, PBMC*
5	Gastric	None	cT3N1M0	pT3N0M0	NA	tumor, two meta-free LNs*, PBMC*

cTNM*: clinical TNM, ypTNM*: post-neoadjuvant therapy pathological TNM, EGJ*: esophagogastric junction cancer, XELOX*: capecitabine and oxaliplatin, FP*: 5-fluorouracil and cisplatin, FOLFOX*: Leucovorin, 5-fluorouracil, and oxaliplatin, meta LN*: metastatic lymph node, meta-free LN*: metastasis-free lymph node, PBMC*: peripheral blood mononuclear cell.

Supplementary Table 2. Gene expression weights in each NMF* component

Gene	NMF0	NMF1	NMF2	NMF3	NMF4	NMF5	NMF6	NMF7	NMF8	NMF9
SELL	0.165	0.407	0.075	0	1.158	0	0	0	0	0.234
PDCD1	0.042	0	0.948	0.713	0	0	0	0.691	0.137	0
TCF7	0	0.059	0.466	0	1.611	0.009	0	0	0.002	0.104
TOX	0.142	0.012	1.070	0.781	0	0	0	0.134	0.294	0.052

NMF*: non-negative matrix factorization

Supplementary Table 3. Characteristics of 55 patients with ICI-naive gastric cancer for flow cytometry analysis.

Age (years)	median (range)	75 (38–90)
Sex	male/ female (%)	41 (74.5)/ 14 (25.5)
Tumor Location	U*/ M*/ L* (%)	22 (40)/ 11 (20) / 22 (40)
Tumor diameter	median (mm, range)	35 (4–100)
Histology	differentiated*/ undifferentiated* (%)	27 (49.1)/ 28 (50.9)
T status	1/ 2/ 3/ 4 (%)	27 (49.1)/ 9 (16.4)/ 6 (10.9)/ 13 (23.6)
N status	0/ 1/ 2/ 3 (%)	28 (50.9)/ 12 (21.8)/ 15 (27.3)
TNM Stage	I/ II/ III (%)	29 (52.7)/ 11 (20.0)/ 15 (27.3)

U*: upper, M*: middle, L*: lower, differentiated*: pap, tub1, tub2, undifferentiated*: por, sig, muc.

Supplementary Table 4. Univariate and multivariate Cox regression analysis for recurrence-free survival in 49 patients with ICI-naive gastric cancer for flow cytometry analysis.

		Univariate			Multivariate		
variable	category	HR*	95% CI*	*P* value	HR*	95% CI*	*P* value
Age (years)	< 75/ ≥ 75	0.428	0.111-1.656	0.219			
Sex	male/ female	0.205	0.058-0.727	0.014	0.221	0.048-1.020	0.053
Tumor location	U*/ M* or L*	1.820	0.521-6.353	0.348			
Tumor diameter (mm)	< 35/ ≥ 35	0.576	0.148-2.252	0.428			
Histology	differentiated*/ undifferentiated*	0.242	0.051-1.142	0.073	0.330	0.032-3.438	0.354
pStage	I/ II or III	0.205	0.043-0.968	0.045	0.234	0.023-2.331	0.216
Tpex/Tex*	< 1.40/ > 1.40	8.646	1.061-70.443	0.044	6.680	0.790-56.492	0.081

HR*: Hazard Ratio, CI*: confidence interval, U*: upper, M*: middle, L*: lower, differentiated*: pap, tub1, tub2, undifferentiated*: por, sig, muc, Tpex/Tex*: The value of Tpex/Tex was calculated as the ratio of $TCF1^{+}PD\text{-}1^{+}$ to $TCF1^{-}PD\text{-}1^{+}$ among $CD8^{+}$ T cells and divided into two groups, above or below the median, respectively. The Wald test was used for univariate and multivariate analysis. Univariate and multivariate Cox regression analyses were performed to estimate hazard ratios (HRs) and 95% confidence intervals. All tests were two-sided. No adjustment for multiple comparisons was performed.

Supplementary Table 5. Clinicopathological characteristics of 14 patients who underwent surgical resection after ICI treatment for flow cytometry analysis.

No	Cancer type	Preoperative ICI treatment (courses)	cTNM*	ypTNM*	Pathological Response
1	Gastric	Nivolumab 24 courses	cT3N3M1 [LYM, PUL]	ypT4aN0M0	Grade 1a
2	Gastric	Nivolumab 93 courses	cT4bN1M1 [HEP]	ypT4aN0M0	Grade 1a
3	EGJ*	SOX* + Nivolumab 3 courses	cT4bN2M1 [LYM]	ypT4aN1M0	Grade 2
4	Esophageal	FP* + Pembrolizumab 2 courses	cT3N1M1 [HEP, LYM]	ypT4bN3M1 [LYM]	Grade 1a
5	Colorectal	Pembrolizumab 6 courses	cT4bN1bM0	ypT0N0M0	Grade 3
6	Esophageal	Nivolumab 7 courses	cT4bN1M0	ypT4bN0M0	Grade 1a
7	Esophageal	FP* + Pembrolizumab 6 courses	cT3N3M1 [HEP]	ypT1aN3M0	Grade 2
8	Esophageal	Nivolumab More than 150 courses	cT4bN2M1 [PUL, HEP]	ypT3N0M0	Grade 1a
9	EGJ*	XELOX* + Nivolumab 3 courses	cT3N1M1 [HEP]	ypT3N2M0	Grade 1a
10	Gastric	SOX* + Nivolumab 6 courses	cT4aN3M1 [HEP]	ypT0N0M0	Grade 3
11	EGJ*	FP* + Pembrolizumab 6 courses	cT3N2M1 [HEP]	ypT2N0M0	Grade 1a
12	Esophageal	FP* + Pembrolizumab 6 courses	cT3N3M1 [HEP]	ypT0N1M0	Grade 3
13	Esophageal	FP* + Pembrolizumab 3 courses	cT3N1M1 [LYM]	ypT0N0M0	Grade 3
14	Esophageal	FP* + Nivolumab 6 courses	cT3N2M1 [LYM]	ypT0N0M0	Grade 3

cTNM*: clinical TNM, ypTNM*: post-neoadjuvant therapy pathological TNM, EGJ*: esophagogastric junction cancer, SOX*: S-1 and oxaliplatin, FP*: 5-fluorouracil and cisplatin, XELOX*: capecitabine and oxaliplatin, TNM status was recorded according to the *Union for International Cancer Control TNM Classification of Malignant Tumors (7th edition)*, pathological response was assessed according to the Japanese Classification of Gastric Carcinoma criteria, LYM: lymph nodes, PUL: pulmonary, HEP: hepatic.

Supplementary Table 6. Frequency of IgG4 binding and non-binding cells among clusters in ICI-treated patients by single-cell RNA/TCR/CITE-seq analysis.

Cluster	IgG4 non-binding	IgG4 binding
C00	0.869521	0.130479
C01	0.656514	0.343486
C02	0.928021	0.071979
**C03**	**0.482096**	**0.517904**
**C04**	**0.039084**	**0.960916**
**C05**	**0.206620**	**0.793380**
**C06.0**	**0.003632**	**0.996368**
**C06.1**	**0.000000**	**1.000000**
**C07**	**0.092086**	**0.907914**
C08	0.560694	0.439306
**C09**	**0.030000**	**0.970000**
C10	0.895849	0.104151
C11	0.863722	0.136278
**C12**	**0.266278**	**0.733722**
C13	0.500836	0.499164
**C14**	**0.431677**	**0.568323**
C15	0.598802	0.401198
C16	0.819549	0.180451
**C17.0**	**0.000000**	**1.000000**
**C17.1**	**0.071429**	**0.928571**
**C17.2**	**0.023810**	**0.976190**
C18	0.504505	0.495495
**C19**	**0.025641**	**0.974359**

IgG4-high clusters (more than 0.50) are in bold.

Supplementary Table 7. Frequency of clonally expanded cells among clusters in ICI-treated patients by single-cell RNA/TCR/CITE-seq analysis.

Cluster	frequency
C00	0.013252
C01	0.106607
C02	0.006684
C03	0.080078
C04	0.881630
C05	0.627884
C06.0	0.935835
C06.1	0.825832
C07	0.484173
C08	0.731936
C09	0.356667
C10	0.023307
C11	0.008459
C12	0.725948
C13	0.057692
C14	0.465839
C15	0.056886
C16	0.477444
C17.0	0.895652
C17.1	0.714286
C17.2	0.547619
C18	0.027027
C19	0.897436
